# Institutional Notes and News

**Published:** 1911-03-25

**Authors:** 


### Institutional Notes and News.

**North Staffordshire Infirmar**

During the year 1910 the whole of the rules of this institution have been thoroughly reviewed, and some very important alterations have been made in them. Amongst other changes it was decided to increase the size of the Honorary Medical Staff by the addition of two assistant physicians, two assistant surgeons, and one additional medical officer for the Electrical Department. Early in this year the committee—in accordance with the powers given them under the rules—appointed Mr. John Russell, who for eleven years had been one of the assistant honorary physicians, and Mr. Reginald Alcock, who for twelve years had been one of the assistant honorary surgeons, to the senior staff. The committee had also decided to set apart a special department for the ear, nose, and throat, and this is now under the charge of Mr. W. C. Allardice, M.D., C.M. Glasgow, F.R.C.S. Edin., the senior honorary assistant surgeon.

**Royal Free, Annual Meeting.**

The presence of Princess Christian, the president, at the annual court of governors of the Royal Free Hospital last week, attracted a large attendance, which included the Bishop of Islington, Sir Edwin Durning Lawrence, Sir Richard Stapley, Mr. R. H. Gamlen (treasurer), Mr. Holroyd Chaplin (Chairman of the Weekly Ward), Mr. James Berry, F.R.C.S., and many others. In moving the adoption of the report, Mr. Chaplin said : I must first remind you that our honoured patron King Edward has passed away, whom many here will remember when as Prince of Wales he opened the Alexandra wing of this hospital. The full measure of work has been done during the year, nearly two-thirds of the patients coming from the immediate neighbourhood, which contains many factories where accidents, if only a small percentage to the numbers employed, furnish numerous cases to the hospital. I would call the attention of Insurance companies and employers to the expenses of surgical treatment. The discharge of their obligations under the Workmen's Compensation Act is far from their sole duty. The gratuitous services of some of the first surgeons in London saves the employers by shortening the time during which the weekly payment has to be made, and saves often the capital sums payable at death. Our ingenuity has been more than ever taxed to find space for growing work. We have had to take another house in Mecklenburgh Square for extra nurses, where we had already one house for maternity work. After searching every corner, space was found in which to put up a very simple structure for patients waiting for the massage treatment that is so necessary after surgery. We have now before us the difficulty of our out-patients' department. All who have seen it will agree with the visitors from the King's Fund that it is inadequate and unsuitable for the many thousands of patients there treated. The first difficulty, about land, I hope may be met. Our request for more land was received favourably by the late Lord Calthorpe, on whose land the hospital was originally built, but the negotiations were temporarily cut short by his death. The Committee hopes that some satisfactory arrangement may be made shortly. I can speak hopefully of our finances. You will see from the report that last year we spent £17,715, and in spite of the generous grant of £4000 from the King's Fund, our ordinary income only gave us £11,000, and for the rest we were fortunately relieved by legacies. We are encouraged by the expectation of receiving the munificent legacy of £50,000 from the late Mr. Silver, who had been an active member of the committee and weekly board for forty years. A chapel is needed, the funds for which are being graciously collected by Princess Marie Louise and the Ladies' Association. It is intended as a memorial to the late King. The board will not be led by the accident of a good year to embark on lavish expenditure in building. The almoners continue to check the possibility of abuse in the out-patient department. The medical school for women continues to flourish, and our relations with it are all the more friendly because it continues to pay its own way. I am sorry to announce that after many years' service our able Secretary, Mr. Conrad Thies, is resigning his post, but we hope to have the advantage of his long experience as a member of the committee of management. After the votes of thanks had been moved the governors and friends were given an opportunity of visiting the wards and other departments.

**Amalgamation Scheme at Bournemouth.**

The recent annual meeting of the Royal Victoria Hospital, Bournemouth, was of wide public interest owing to the adoption of a scheme for its amalgamation with the Royal Boscombe and West Hants Hospital. The approval of this meeting leaves only the agreement of the Boscombe Hospital to be obtained for the amalgamation to be accomplished. The following is an excerpt from the general statement of the committee : "It will be remembered that several times in the history of the hospital efforts have been made in this direction (amalgamation). A special committee, consisting of ten members from each hospital, was appointed to prepare a scheme. As a result of their labours, a set of rules, which, it is hoped, contains a basis for the most satisfactory working of both institutions as a united hospital, was prepared. These rules have been adopted by the general committees of both institutions, and now only await adoption by the Governors. It is hoped that a considerable saving will be effected in administration. All overlapping, either as regards work or equipment, will be prevented, and the linking up of the general medical work in the borough under one board must tend towards increased efficiency. . . . Our institution would form a part of the united hospital, which would be known as the Royal Victoria and West Hants Hospital, Bournemouth. No loss of continuity would result, either in the work or administration, and it is hoped that an increased interest in the town hospital would be created, and a substantial augmentation of financial support follow." Dr. J. Roberts Thompson, chairman, then read the report of the preliminary conference of the two institutions, which contained the following : "We suggest a provisional committee of twenty members, ten from the supporters of each hospital, which shall prepare a set of rules and regulations for the administration of the united hospital. During these deliberations of the Provisional Committee, any matters upon which agreement cannot be arrived at shall be submitted to some hospital expert for his opinion. As one of the main objects of this proposed union is to provide that at some not remote date there shall be one centralised large general hospital for the borough and district, with subsidiary special departments, it is understood that when additional ward accommodation has to be provided, it shall not necessarily be by the extension of any existing buildings, but may be by the erection of a new hospital. In this event, the buildings now existing would be devoted to the work and development of special departments." The Earl of Malmesbury, the hospital's president, congratulated Dr. Roberts Thompson on the successful result of the negotiations.

**Homœopathic Hospital an Queen-Mother.**

The Annual Meeting of the London Homœopathic Hospital was held on Friday last week, Mr. John Pakenham Stilwell, J.P., chairman of the Board of Management, presiding. The following letter was read on behalf of Queen Alexandra from Colonel Sir Arthur Davidson: "Lord Knollys has handed me your letter of March 11, inquiring whether it would be possible for Queen Alexandra to open the new wing of the London Homœopathic Hospital in June. There is, I am afraid, no possible chance of this taking place as, owing to her deep mourning, her Majesty will not take part at present in any public ceremonials. Queen Alexandra takes the deepest interest in all hospitals and their work, and her Majesty has been very interested to see the fine results achieved by the labours and generosity of Sir Henry Tyler and his friends in raising the large sum required for building the new extension of the hospital." This extension, which has increased the number of the hospital's beds from 104 to 166, was viewed by many of those who attended the meeting. The out-patient department has also been extended, and we regret that want of space alone prevents us from going into further details this week. It is satisfactory to note that the hospital has now a capital of £80,000. A new nurses' home is badly wanted, and an appeal is issued for £13,000, at which the cost is estimated.

**Efforts at Colchester.**

A FURTHER appeal has been made by the Governors of the Essex County Hospital, and readers who remember our account of November 12 of a meeting held in its support will not be surprised at this decision. At the recent annual board the sale of Wix Farm was mentioned, and it is due to this and to the efforts of the King Edward Memorial Fund that the deficit of £4,000 has been paid off. The year's working, however, still costs £1,800 more than the hospital's supporters provide for, and in reference to this the Chairman gave some useful figures which very aptly summarise the hospital's work. "We have," he said, "a hospital with 100 beds; we admit 1,000 in-patients and 3,000 out-patients during the year, and we have large premises to keep up. In addition to an honorary staff of seven, 51 persons are employed at the hospital, and of these 28 are resident." This gives a very good idea in brief of what the running of a hospital means. As regards new methods of raising money, the only effort that was clearly enunciated at the meeting was an arrangement for a general canvass of the town. Sir Henry Burdett's suggestion for the formation of a hospital association, which has so often proved its value, does not appear to have been taken up. The Rev. E. Spurrier and Mr. E. A. Blaxhill, the deputy mayor, still have a chance of pushing it forward, or at least of providing new tactics to supplement the efforts of the past.

**Sussex County Hospital Honoured.**

THE Royal Sussex County Hospital must be congratulated upon the title which His Majesty the King has now commanded it to use. This royal recognition should also encourage the hospital's subscribers to remove that £3,500 deficit on last year's expenditure, which, after all the available legacies had been expended, still remained unmet. No doubt one reason why the hospital has been promoted to the rank of a royal institution is the existence of two separate funds for the destination of legacies. One of these, as Mr. Borradaile remarked a week or two ago at the Annual Meeting, is for the permanent endowment of the hospital, the income alone being at the disposal of the committee; the other is known as "the available fund," of which capital as well as income can be dealt with. The subscriber has the choice of leaving a legacy ear-marked to either fund. In the twenty years of Mr. Borradaile's experience the endowment fund has nearly doubled the £50,000 at which it stood in 1890. That is a fine record, but the royal title is only the latest example of the regard which our Sovereigns have felt for this institution. From the visit of William IV. and Queen Adelaide in 1833 to his late Majesty's laying of the foundation-stone of the new out-patient department, when Prince of Wales, in 1896, the hospital has never been without the active support of royalty.

**Bristol Children's Hospital.**

THE new out-patient department that is needed at the Bristol Royal Hospital for Sick Children is dependent on a fund that does not grow as quickly as it ought. The sum of money at present received, £1,541, is not sufficient to justify the committee in beginning to build, and some of the credit for raising this amount belongs to the Ladies' Auxiliary Association. It is true the association aims at the support of the hospital in general, but its support has been so good as surely to have stimulated many to subscribe to one or another of the needs of the hospital. One cot already has been endowed by it in perpetuity, and Miss Parry's efforts have become historic. Mr. I. E. Smith, the secretary, and Mr. Garnett, the treasurer, both impressed on the subscribers the "impending necessity" of building a new out-patient department. The Coronation year should not be allowed to pass without strenuous efforts having been made to raise the necessary money. The Sheriff of Bristol, Mr. Riseley, spoke of amalgamation as a possibility of hospital policy in the future, expressing his opinion as to the saving in energy and money this would cause. Dr. Bertram Rogers approved of a central hospital board, which might grow out of the proposed Sunday fund. He added that the advantages of the hospital's site on the top of a hill were limited by the difficulties some parents felt in getting them there. Babies rather than older children were carried up it.

**Kent County Ophthalmic Hospital.**

THE unexpected discovery of faulty drainage, and the prompt and experienced steps which the authorities of the Kent County Ophthalmic Hospital were compelled to take last year, threw a great burden upon the institution. The improvements have been carried out by the hospital's honorary architect, Mr. Hubert Benstead, under the supervision of the borough's sanitary inspector. But progress has not stopped here, for £75 is to be spent upon an up-to-date radiator system, which will add to the comforts, one might almost say, destroy the former privations, of the out-patients when waiting in winter for their turn. These out-patients continue to increase in numbers, and now crowd out, as Mr Monckton, the chairman, remarked, the central hall. Some £500 is needed to pay off debts, towards which £150 has been received. The Mayor of Maidstone, Councillor F. E. Wallis, has appealed for a King Edward Memorial Fund, and it is hoped that by it both the West Kent Hospital and the Ophthalmic Hospital will benefit. The total number of patients in the wards has been 353, and 8,356 have been received in the out-patient department. The Mayor's attitude has, no doubt, been influenced by those who feel that the question of the amalgamation of the Ophthalmic with the General Hospital must be considered one day as well worth deciding. Amalgamations, however, though becoming increasingly frequent, every city now having to consider them, are thorny questions which affect too many interests to make either party eager to initiate a joint scheme for carrying them out.

